# β-1,3-Glucan Recognition Protein Can Inhibit the Proliferation of *Bombyx mori* Cytoplasmic Polyhedrosis Virus

**DOI:** 10.3390/insects16040431

**Published:** 2025-04-19

**Authors:** Yinong Zhang, Jiming Yan, Yukai Xie, Xiong Wang, Feifei Ren, Haixu Bian, Jingchen Sun

**Affiliations:** 1 Subtropical Sericulture and Mulberry Resources Protection and Safety Engineering Research Center, Guangdong Provincial Key Laboratory of Agro-Animal Genomics and Molecular Breeding, College of Animal Science, South China Agricultural University, Guangzhou 510642, China; yinongzh@foxmail.com (Y.Z.); gmyjm97@sina.com (J.Y.); xieyukai2022@163.com (Y.X.); cyfz@scau.edu.cn (J.S.); 2 College of Veterinary Medicine, South China Agricultural University, Guangzhou 510642, China; 3 College of Life Sciences, Nanyang Normal University, Nanyang 473061, China; wangttx@outlook.com; 4 Department of Microbiology, College of Preclinical Medicine, Zunyi Medical University, Zunyi 563009, China

**Keywords:** βGRP-3, innate immune, *Bombyx mori*, BmCPV

## Abstract

Insects detect pathogens through their germ-line encoded pattern recognition receptors (PRRs). Among these, β-1,3-glucan recognition protein (βGRP) is a crucial PRR that specifically identifies pathogenic microorganisms and triggers innate immune signaling cascades. However, it remains unclear whether βGRP can detect viruses and protect the host from viral threats. In this study, using high-throughput sequencing technology, we observed a significant suppression of βGRP-3 in *Bombyx mori* during infection with the *Bombyx mori* cytoplasmic polyhedrosis virus (BmCPV). Moreover, overexpression of βGRP-3 in BmN cell lines resulted in a reduction of BmCPV proliferation, whereas knockdown of βGRP-3 in BmN cells promoted BmCPV proliferation. These findings suggest that the βGRP family functions not only as anti-bacterial, antifungal, and anti-yeast PRRs but also as protectors against various harmful viruses in insects.

## Simple Summary

Insects utilize β-1,3-glucan recognition proteins (βGRPs) to detect pathogens and activate immune responses. This study reveals that βGRP-3 in *Bombyx mori* confers protection against BmCPV, a non-enveloped RNA virus, as its overexpression inhibits viral replication while knockdown enhances it. These findings demonstrate that βGRPs not only combat bacteria and fungi but also play a role in antiviral defense, broadening their immunological functions.

## 1. Introduction

In the course of a billion years of evolution, animals have confronted the formidable challenges posed by pathogens. Among these, invertebrates, notably insects, have adeptly relied upon their innate immune system to defend against threats from parasites, bacteria, fungi, and viruses. In their pursuit of optimal survival strategies, insects sense pathogen-associated molecular patterns (PAMPs) using their germ-line encoded pattern recognition receptors (PRRs). These precise recognitions ensure the timely initiation of downstream innate immune responses, enhancing the ability of insects to combat and overcome the diverse array of pathogens encountered.

As one of the most critical pattern recognition receptors (PRRs) in insect immune defense against microbial invasions, β-1,3-glucan recognition protein (βGRP) specifically binds to β-1,3-glucan and activates downstream immune signaling cascades [1,2,3]. Structurally, βGRP comprises two conserved functional domains: an N-terminal domain homologous to gram-negative bacteria-binding protein (GNBP) [4,5] and a C-terminal domain resembling laminarinase [6,7,8,9,10,11,12,13,14,15]. The N-terminal domain interacts with triple-helical β-glucan, initiating the prophenoloxidase (proPO) cascade and Toll pathway [16], while the function of the C-terminal domain remains poorly understood [17]. Through the specific interaction between the N-terminal domain of βGRP and β-1,3-glucans, insects can recognize invasive fungi, bacteria, and yeast via the β-1,3-glucans present on their cell walls [6,9,10,18,19]. This broad-spectrum recognition capability establishes the βGRP family as a critical defense mechanism against a wide range of microorganisms.

Traditionally, viruses have not been considered primary targets of βGRP-mediated immunity due to the absence of β-1,3-glucans in their structure. However, recent studies have revealed that BmβGRP-4 can inhibit the proliferation of *Bombyx mori* nucleopolyhedrovirus (BmNPV), a large enveloped DNA virus, by inducing apoptosis [20]. This finding provides new insights into the immune functions of the βGRP family, suggesting that βGRPs may recognize viral components other than β-1,3-glucans. Consequently, βGRP emerges as a potential antiviral gene and a broad-spectrum immune target. Nevertheless, further research is required to elucidate the antiviral mechanisms of βGRP, particularly against diverse virus types, including RNA viruses and non-enveloped viruses, due to the limited data available.

In this study, using high-throughput sequencing technology, we observed a significant downregulation of βGRP-3 in *Bombyx mori* during infection with the *Bombyx mori* cytoplasmic polyhedrosis virus (BmCPV), a non-enveloped virus characterized by its segmented double-stranded RNA (dsRNA) genome. This finding suggests a potential role for βGRP-3 in viral infection. To investigate this hypothesis, we overexpressed βGRP-3 in the BmN cell line and conducted BmCPV infection assays. The results demonstrated that βGRP-3 overexpression significantly reduced BmCPV proliferation in BmN cells. Conversely, knockdown of βGRP-3 facilitated viral proliferation. These findings indicate that the βGRP family not only serves as a PRR against bacteria, fungi, and yeast but also plays a protective role in defending insects against diverse viral pathogens.

## 2. Material and Methods

### 2.1. Silkworms, Cell Culture, and Virus Propagation

The Dazao P50 strain of silkworm larvae, the *Bombyx mori* ovary-derived BmN cell line, and the *Bombyx mori* cytoplasmic polyhedrosis virus type 1 (BmCPV-1) polyhedra were maintained under controlled laboratory conditions. Silkworm larvae were reared on a diet of fresh mulberry leaves at a constant temperature of 25 °C and a relative humidity of 70–80%. The BmN cell line was cultured at 28 °C in Grace’s insect medium (Gibco, Waltham, MA, USA), supplemented with 10% fetal bovine serum (FBS; Gibco, USA) to support optimal growth. BmCPV polyhedra were propagated and subsequently purified from the midgut tissues of fifth-instar silkworm larvae.

### 2.2. Tissue Sample Collection for RNA-Seq Analysis

Newly molted fifth-instar silkworm larvae were orally inoculated with BmCPV-1 polyhedra suspended in phosphate-buffered saline (PBS) at a concentration of 6 × 10^6^ polyhedra per larva, administered using a microliter syringe. Larvae in the control group were administered an equivalent volume of PBS without polyhedra. Following inoculation, all larvae were provided with fresh mulberry leaves ad libitum. At 96 h post-infection (h.p.i.), midgut tissues were dissected and collected from larvae in both the infected and control groups. Three biological replicates were prepared for each group, with each replicate comprising pooled midgut tissues from 10 larvae. This resulted in a total of six samples: three infected midgut samples (designated as BmCPV-MG-1, BmCPV-MG-2, and BmCPV-MG-3) and three control midgut samples (designated as NC-MG-1, NC-MG-2, and NC-MG-3).

### 2.3. RNA Extraction and RNA-Seq Analysis

Total RNA was extracted from the six midgut samples using Trizol reagent (Invitrogen, Waltham, MA, USA) [21]. RNA integrity and quality were assessed using an Agilent 2100 Bioanalyzer (Agilent Technologies, Santa Clara, CA, USA). Messenger RNA (mRNA) was enriched using Oligo (dT) beads and subsequently fragmented using a fragmentation buffer. The fragmented mRNA was reverse-transcribed into complementary DNA (cDNA) using the NEBNext Ultra RNA Library Prep Kit for Illumina (New England Biolabs, Ipswich, MA, USA). Double-stranded cDNA fragments were purified using the QiaQuick PCR extraction kit (Qiagen, Hilden, Germany). The purified cDNA fragments were subjected to end repair, poly (A) tail addition, and ligation to Illumina sequencing adapters. The ligation products were purified using AMPure XP Beads (1.0×), followed by size selection via agarose gel electrophoresis. The resulting cDNA libraries were amplified by polymerase chain reaction (PCR) and sequenced on the Illumina Novaseq6000 platform by Gene Denovo Biotechnology Co. (Guangzhou, China) to generate high-throughput sequencing data for downstream analysis.

Raw sequencing reads were processed to obtain clean reads using fastp (v0.18.0) [22], which included the removal of low-quality reads and adapter sequences. Ribosomal RNA (rRNA) sequences were further eliminated using Bowtie2 (v2.2.8) [23]. The remaining high-quality reads were aligned to the latest version of the *Bombyx mori* genome (genome sequences and annotation files were retrieved from SilkDB 3.0, https://silkdb.bioinfotoolkits.net/doc/download.html (accessed on 25 July 2023)) using HISAT2 (v2.4) [24]. The aligned reads for each sample were assembled into transcripts using StringTie (v1.3.1) [25]. Gene abundance was quantified using the RNA-seq by Expectation Maximization (RSEM) software [26], and gene expression levels were normalized using the Fragments Per Kilobase of transcript per Million mapped reads (FPKM) method.

Differential gene expression analysis between the control (NC-MG) and BmCPV-infected (BmCPV-MG) groups was performed using DESeq2 software [27]. Genes with a false discovery rate (FDR) ≤ 0.05 and an absolute fold change of |log2FC| > 1 were identified as differentially expressed genes (DEGs). Functional annotation and pathway enrichment analyses of the DEGs were conducted using Gene Ontology (GO) and the Kyoto Encyclopedia of Genes and Genomes (KEGG) databases [28,29].

### 2.4. Quantitative Real-Time PCR (qPCR)

Total RNA was extracted from tissue and cell samples using Trizol reagent (Invitrogen, USA) [21]. Reverse transcription was performed using the PrimeScript RT Reagent Kit with gDNA Eraser (TaKaRa, Dalian, China). Quantitative real-time PCR was conducted on a CFX96 PCR System (Bio-Rad, Hercules, CA, USA) equipped with Bio-Rad CFX Manager Software (v3.1). Gene expression levels were quantified using SYBR green (Bio-Rad, USA). Primer sequences for qPCR are listed in Table 1, and each reaction was performed in triplicate. Relative gene expression was calculated using the 2^−ΔCT^ (Livak) method, with BmRP49 (*Bombyx mori* ribosomal protein 49) serving as the internal reference gene [30].

### 2.5. Construction of Overexpression Plasmids

The pIZT/V5-egfp vector was used as the backbone for constructing the *BmβGRP-3* overexpression plasmid. The open reading frame (ORF) of *BmβGRP-3*, flanked by 20–25 bp of vector homology arms at both ends, was amplified from a silkworm midgut cDNA library. The amplified fragment was inserted into an Xba I-digested linearized pIZT/V5-egfp plasmid using the Seamless Cloning Kit (Beyotime, Haimen, China), resulting in the pIZT-βGRP-egfp recombinant plasmid. Additionally, an mCherry fragment was inserted into the Xba I-digested pIZT/V5-egfp plasmid to generate the pIZT-mCherry-egfp control plasmid. The primer sequences used for cloning are provided in Table 2.

### 2.6. dsRNA Synthesis

Double-stranded RNA (dsRNA) was synthesized in vitro using the T7 RiboMAX Express RNAi System (Promega, Madison, WI, USA) according to the manufacturer’s instructions. Primer sequences for amplifying target gene fragments with T7 RNA polymerase promoters are listed in Table 2.

### 2.7. Cell Transfection

BmN cells were seeded in a 24-well plate (Thermo, Waltham, MA, USA) and cultured until reaching 60–80% confluency. Cells were washed with serum-free Grace’s medium and transfected with either overexpression plasmids or dsRNA using FuGENE HD Transfection Reagent (Promega, Madison, WI, USA) following the manufacturer’s protocol. At 6 h post-transfection, the medium was replaced with fresh Grace’s medium supplemented with serum.

### 2.8. BmCPV Virion Purification

BmCPV virions were purified as previously described [31,32]. Briefly, BmCPV polyhedra were treated with 0.2 M 0.2 M Na_2_CO_3_–NaHCO_3_ buffer (pH 10.8) for 60 min, followed by centrifugation at 10,000× *g* for 40 min to remove debris. The supernatant was further centrifuged at 80,000× *g* for 60 min to pellet the virions. The resulting pellet was resuspended in serum-free Grace’s medium for subsequent infection assays.

### 2.9. Overexpression of BmβGRP-3 and BmCPV Infection in BmN Cells

BmN cells were transfected with 500 ng/well of either pIZT-βGRP-egfp or pIZT-mCherry-egfp (control). Protein samples were collected at 24, 48, and 72 h post-transfection, and *BmβGRP-3* expression was assessed by Western blotting. At 24 h post-transfection, the medium was replaced with serum-free Grace’s medium (Gibco, USA) containing purified BmCPV virions (~1 × 10^6^ polyhedra per well). After 6 h of infection, cells were washed three times with PBS and incubated in fresh serum-containing Grace’s medium for further analysis.

### 2.10. Construction of BmβGRP-3 Stable Overexpression Cell Line (BmN-βGRP)

BmN cells were transfected with 500 ng/well of either pIZT-βGRP-egfp or pIZT-mCherry-egfp (control). Upon observing green fluorescence, the medium was replaced with Grace’s medium containing 0.3% (*v*/*v*) bleomycin to select cells carrying the bleomycin resistance gene (encoded by the pIZT/V5 plasmid). The bleomycin-containing medium was refreshed every five days until cells reached 60–80% confluency. Selected cells were subcultured for six generations, and *BmβGRP-3* expression was confirmed to establish the BmN-βGRP stable cell line.

### 2.11. RNA Interference (RNAi) of BmβGRP-3 and BmCPV Infection

BmN-βGRP cells were transfected with varying concentrations of dsRNA-βGRP or dsRNA-Red (1, 3, and 5 μg/well) to determine the optimal concentration, with samples collected at 24 h post-transfection. dsRNA-Red served as the control. Subsequently, cells were transfected with the optimal dsRNA concentration, and samples were collected at 24, 48, 72, and 96 h post-transfection to assess *BmβGRP-3* expression by qPCR. At 24 h post-transfection, cells were infected with BmCPV virions as described above.

### 2.12. Western Blotting Analysis

Total protein was extracted using RIPA buffer (Beyotime, China), and protein concentrations were determined using the BCA Protein Assay Kit (Beyotime, China). BmCPV VP1 protein was detected using a mouse polyclonal antibody (prepared and preserved in the laboratory) and an HRP-labeled goat anti-mouse IgG (H + L) secondary antibody (Beyotime, China). *BmβGRP-3* was detected using a mouse monoclonal antibody specific for the V5 tag (Invitrogen, USA), with alpha-tubulin (Beyotime, China) serving as the loading control. Protein bands were visualized using a ChemiDoc XRS+ Chemiluminescent Imaging System (Bio-Rad, USA).

### 2.13. Statistical Analysis

Statistical analyses were performed using GraphPad Prism (v9.0.0). Two-way ANOVA and *t*-tests were used to determine significant differences (* *p* < 0.05, ** *p* < 0.01, *** *p* < 0.001, and **** *p* < 0.0001). Data are presented as the mean ± standard deviation (SD) from three independent experiments.

## 3. Result

### 3.1. RNA-Seq, Data Analysis, and Validation

**Figure 1:**
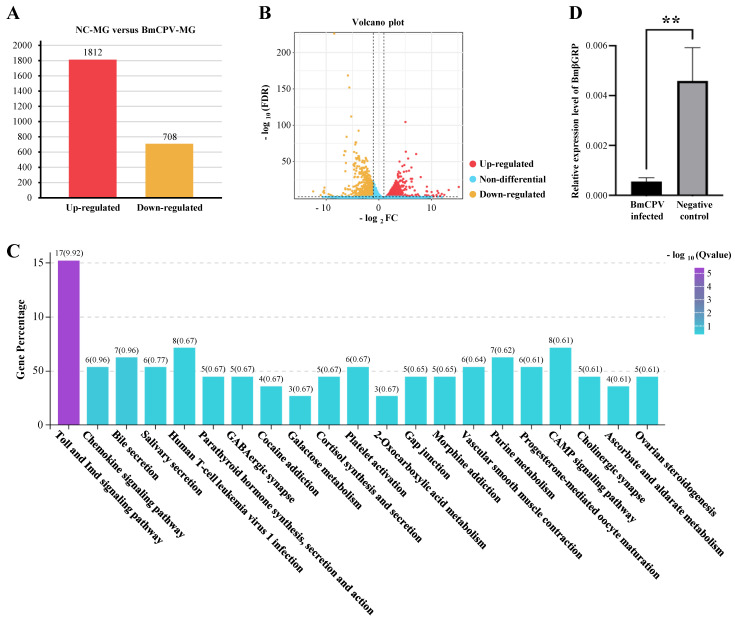
RNA-Seq Analysis of Midgut Samples of silkworm larvae feed with pure PBS (NC-MG) or BmCPV polyhedra suspended PBS (BmCPV-MG). (**A**) Statistics of Differentially Expressed Genes (DEGs) in the *Bombyx mori* midgut after BmCPV infection shown in a bar chart. (**B**) Volcano plot illustrating the distribution of DEGs after BmCPV infection. (**C**) KEGG pathway enrichment analysis of pathways in BmN cells after BmCPV infection. (**D**) Validation of RNA-Seq data by qPCR for BmβGRP. Data are presented as mean ± SD from three biologically independent experiments. ** *p* < 0.01.

Our previous study has shown the detailed DEG analysis results of RNA-seq Data [33]. In brief, total RNA were extracted from midgut tissue of 3 BmCPV-infected *Bombyx mori* larva and 3 *Bombyx mori* larva control, and then reverse transcript into the 6 cDNA libraries with universal primers. Six cDNA libraries were sequenced and produced 43,837,023,900 bp of raw data and 291,239,754 clean reads. Clean reads were then mapped onto the silkworm genome database (SilkDB 3.0, https://silkdb.bioinfotoolkits.net/doc/download.html (accessed on 25 July 2023)). Compared to the uninfected *Bombyx mori* larva control, we identified 2520 differentially expressed genes (DEGs), including 1812 up-regulated DEGs and 708 down regulated DEGs after BmCPV infection (Figure 1A,B and  Figure S1). KEGG pathway enrichment analysis shown that the Toll and Imd signaling pathway of *Bombyx mori* was significantly regulated after the BmCPV infection (Figure 1C and Figure S2), indicate that the upstream genes of these pathway, such as the BmpGRP and BmβGRP, might have function to sense the viral infection [33]. Within the RNA-seq data, the immune-relevant gene *BmβGRP-3* (accession number in SilkDB 3.0: BMSK0006299), previously thought to have antibacterial and antifungal functions but not antiviral activity, was significantly suppressed. We, therefore, propose that BmβGRP may possess an unidentified antiviral function. To investigate whether BmβGRP has antiviral properties or if this suppression is merely an anomaly, we first used q-PCR to measure the expression levels of BmβGRP in both BmCPV infected and uninfected silkworm’s midgut sample. The results showed that, 96 h after feeding BmCPV polyhedra to the silkworms, BmβGRP expression was significantly suppressed in the midgut, consistent with the RNA-seq data (Figure 1D).

### 3.2. Overexpression of BmβGRP Suppresses BmCPV Proliferation in BmN Cells

**Figure 2:**
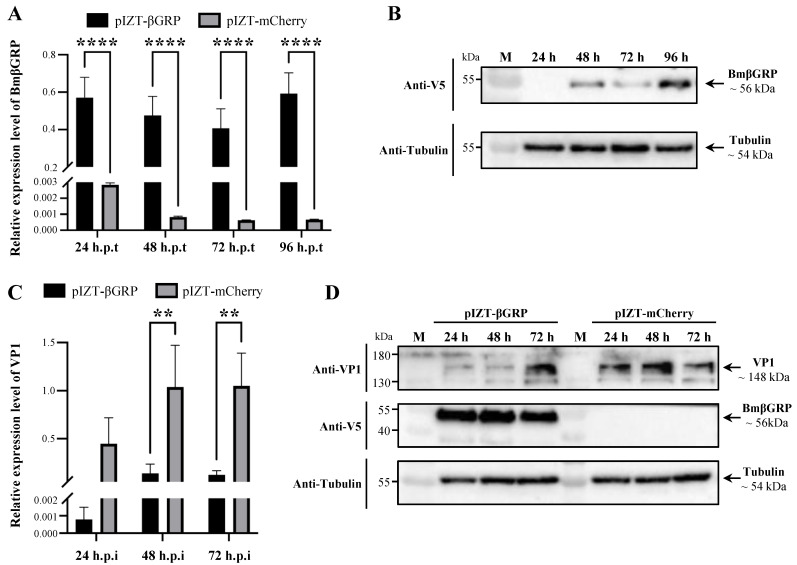
Transient Overexpression of BmβGRP Inhibits BmCPV Proliferation in BmN Cells. (**A**) Relative expression levels of the BmβGRP gene detected in BmN cells transfected with pIZT-βGRP or pIZT-mCherry by qPCR at 24, 48, 72, and 96 h post-transfection (h.p.t). Data represent mean ± SD from three biologically independent experiments. **** *p* < 0.0001. (**B**) Expression levels of BmβGRP protein detected in BmN cells transfected with pIZT-βGRP or pIZT-mCherry by western blot analysis at 24, 48, 72, and 96 h.p.t. (**C**) Relative expression levels of the BmCPV VP1 gene detected in BmCPV-infected BmN cells transfected with pIZT-βGRP or pIZT-mCherry by qPCR at 24, 48, and 72 h post-infection (h.p.i). BmN cells were transfected with pIZT-βGRP or pIZT-mCherry 48 h prior to BmCPV infection. Data represent mean ± SD from three biologically independent experiments. ** *p* < 0.01. (**D**) Expression levels of BmCPV VP1 protein detected in BmCPV-infected BmN cells transfected with pIZT-βGRP or pIZT-mCherry by western blot analysis at 24, 48, and 72 h.p.i. BmN cells were transfected with pIZT-βGRP or pIZT-mCherry 48 h prior to BmCPV infection.

In order to investigate the impact of BmβGRP on BmCPV proliferation, we transiently transfected BmN cells with pIZT/V5-BmβGRP and pIZT/V5-mCherry vectors to obtain BmN-tβGRP cells expressing BmβGRP and BmN-tmCherry control cells, respectively. The expression levels of the BmβGRP gene in these cells were detected at various time points post-transfection using q-PCR. The results showed a significant upregulation of BmβGRP gene expression in BmN-tβGRP cells compared to BmN-tmCherry cells at 24, 48, 72, and 96 h post-plasmid transfection (Figure 2A). However, Western Blot analysis revealed detectable expression of BmβGRP protein in BmN-tβGRP cells only after 48 h of transfection with the overexpression vector (Figure 2B). Therefore, for subsequent experiments, we chose to conduct BmCPV infection assays 48 h post-transfection with the overexpression vector.

After 48 h of transfection with the overexpression vector, purified BmCPV viral particles were used to infect both BmN-tβGRP and BmN-tmCherry cells. The expression levels of the BmCPV VP1 gene were determined by q-PCR at 24, 48, and 72 h post-infection. The results showed significantly lower expression levels of the VP1 gene in BmN-tβGRP cells compared to BmN-tmCherry cells at 48 and 72 h post-infection (Figure 2C). Western Blot analysis also revealed significantly lower expression levels of the VP1 protein in BmN-tβGRP cells compared to BmN-tmCherry cells at 24 and 48 h post-infection (Figure 2D). These findings indicate that BmβGRP can suppress the proliferation of BmCPV in cells.

### 3.3. Stable Overexpression of BmβGRP in BmN Cells Reduces Early Intracellular Proliferation of BmCPV Virus

**Figure 3:**
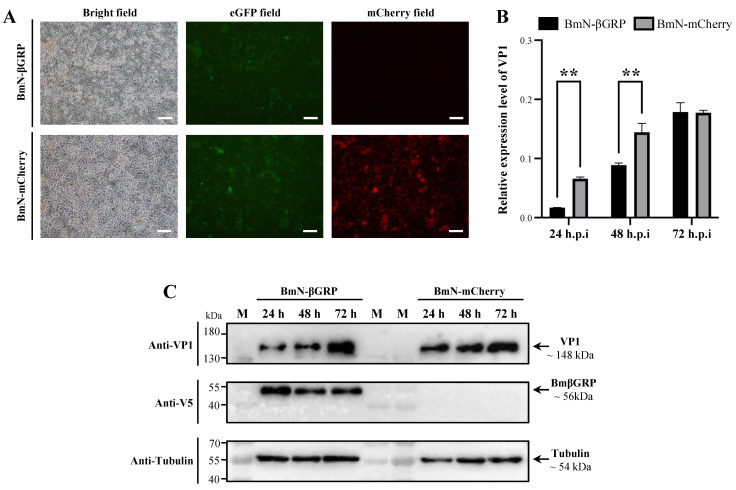
Stable Overexpression of BmβGRP Inhibits BmCPV Proliferation in BmN Cells. (**A**) Observation of constructed BmN cells stably overexpressing BmβGRP (BmN-βGRP) and mCherry (BmN-mCherry) under a fluorescence microscope equipped with a Nikon B-2A filter (EX: 450–490, DM: 505, BA: 520 for eGFP field) and Nikon G-2A filter (EX: 510–560, DM: 575, BA: 590 for mCherry field). Scale bar = 100 μm. (**B**) Relative expression level of BmCPV VP1 gene detected in BmCPV-infected BmN-βGRP and BmN-mCherry cells by qPCR at 24, 48, and 72 h.p.i. Data represent mean ± SD from three biologically independent experiments. ** *p* < 0.01. (**C**) Expression level of BmCPV VP1 protein detected in BmCPV-infected BmN-βGRP and BmN-mCherry cells by western blot analysis at 24, 48, and 72 h.p.i.

To further validate the influence of BmβGRP on the intracellular proliferation of the BmCPV virus, we established BmN cell lines that can stably overexpress BmβGRP. The BmN cell lines were transfected with the pIZT/V5-βGRP vector. Following antibiotic selection pressure (bleomycin) and passage to the fifth generation (P5) while still maintaining fluorescence, stable expression of BmβGRP protein was confirmed in the P5 generation (Figure 3A). Therefore, we successfully constructed a stable BmN-βGRP cell line expressing BmβGRP. Similarly, we constructed a stable BmN-mCherry cell line expressing mCherry using the pIZT/V5-mCherry vector (Figure 3A). Both cell lines were subjected to BmCPV infection assays to further validate the antiviral function of BmβGRP against BmCPV.

Purified BmCPV virions were used to infect BmN-βGRP and BmN-mCherry cells, and the expression levels of the BmCPV VP1 gene were detected by qPCR at 24, 48, and 72 h post-infection. The results showed that at 24 and 48 h post-infection, the expression levels of the BmCPV VP1 gene in BmN-βGRP cells were significantly lower than those in BmN-mCherry cells, while no significant difference was observed at 72 h post-infection (Figure 3B). Western blot analysis revealed a similar trend in the expression levels of the VP1 protein, with significantly lower levels observed in BmN-βGRP cells at 24 and 48 h post-infection compared to BmN-mCherry cells, with no significant difference observed at 72 h post-infection (Figure 3C). These results indicate that BmβGRP can inhibit the intracellular proliferation of the BmCPV virus during the early stages of infection.

### 3.4. Knock Down of BmβGRP Promotes BmCPV Proliferation in BmN Cells

**Figure 4:**
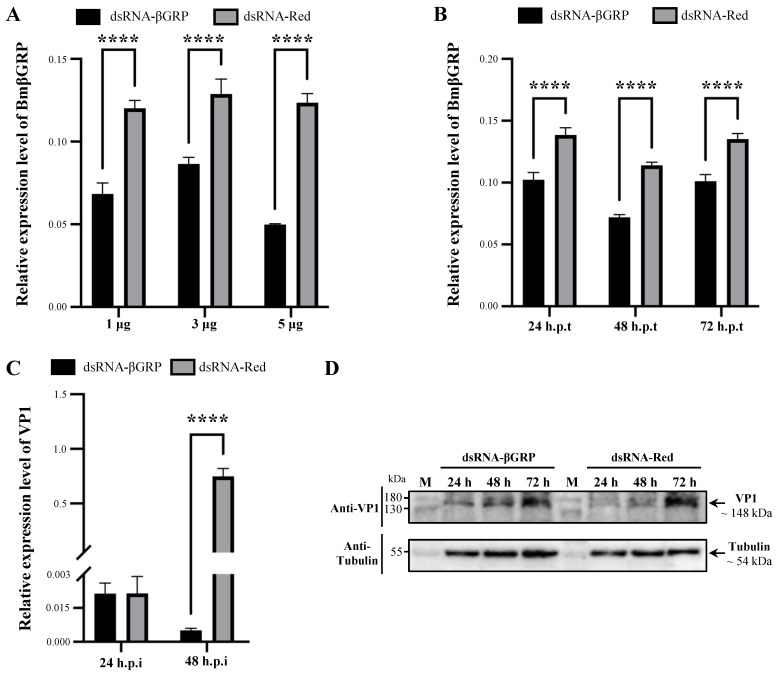
Knockdown of the BmβGRP Gene in BmN-βGRP Cells by RNA Interference (RNAi) Promotes BmCPV Proliferation. (**A**) Relative expression level of the BmβGRP gene detected in BmN-βGRP cells transfected with 1, 3, and 5 μg dsRNA-βGRP or dsRNA-Red by qPCR. Data represent mean ± SD from three biologically independent experiments. **** *p* < 0.0001. (**B**) Relative expression level of the BmβGRP gene detected in BmN-βGRP cells transfected with 5 μg dsRNA-βGRP or dsRNA-Red by qPCR at 24, 48, and 72 h.p.t. Data represent mean ± SD from three biologically independent experiments. **** *p* < 0.0001. (**C**) Relative expression level of the BmCPV VP1 gene detected in BmCPV-infected BmN-βGRP cells (transfected with dsRNA) by qPCR at 24, 48, and 72 h.p.i. The BmN-βGRP cells were transfected with 5 μg dsRNA-βGRP or dsRNA-Red 48 h prior to BmCPV infection. Data represent mean ± SD from three biologically independent experiments. **** *p* < 0.0001. (**D**) Expression level of the BmCPV VP1 protein detected in BmCPV-infected BmN-βGRP cells (transfected with dsRNA) by western blot analysis at 24, 48, and 72 h.p.i. The BmN-βGRP cells were transfected with 5 μg dsRNA-βGRP or dsRNA-Red 48 h prior to BmCPV infection.

To further verify the impact of BmβGRP on intracellular proliferation of BmCPV virus, we attempted to reduce the expression of BmβGRP in BmN cells through dsRNA interference. However, the results showed that direct RNA interference in BmN cells did not stably reduce the expression of BmβGRP. Therefore, we tried to knock down the expression of BmβGRP by transfecting BmN-βGRP cell lines with dsRNA at different concentrations. q-PCR results indicated that dsRNA at concentrations of 1, 3, and 5 μg effectively reduced the expression of the BmβGRP gene in BmN-βGRP cell lines, with the interference effect being most pronounced with 5 μg of dsRNA (Figure 4A). Subsequently, we detected the expression of BmβGRP at 24, 48, and 72 h post-transfection and found that the interference efficiency was highest at 48 h post-transfection (Figure 4B). Therefore, we chose to conduct BmCPV virus challenge experiments 48 h after transfection with 5 μg of dsRNA in BmN-βGRP cell lines.

We infected BmN-βGRP cell lines transfected with dsRNA-βGRP or dsRNA-Red with BmCPV virus particles separately and used q-PCR to detect the expression levels of the virus VP1 gene at 24 and 48 h post-infection. The results showed that at 48 h post-infection, the expression levels of the virus VP1 gene in cell lines transfected with dsRNA-βGRP were significantly higher compared to those transfected with dsRNA-Red (Figure 4C). Western blot analysis also revealed a significant increase in the expression levels of the virus VP1 protein in cell lines transfected with dsRNA-βGRP at 24- and 48-h post-infection compared to those transfected with dsRNA-Red (Figure 4D). These results indicate that knocking down the BmβGRP gene can promote the proliferation of the BmCPV virus in cells.

### 3.5. BmβGRP Can Activate the ProPO Cascade, Toll Pathway, and Relish-Mediated Immune Cascade

**Figure 5:**
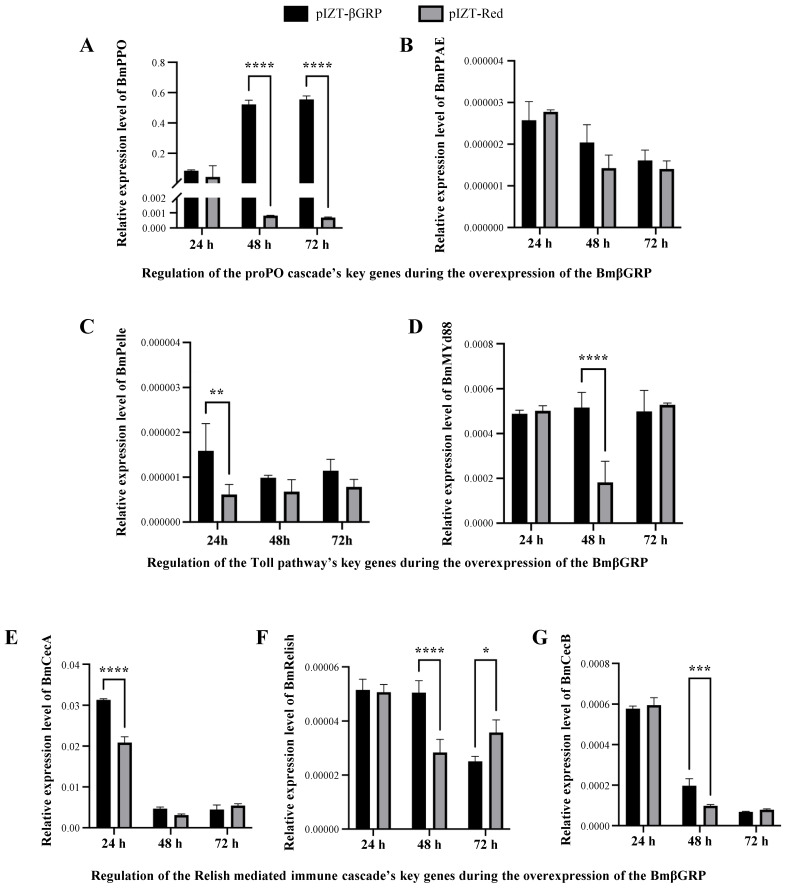
Regulation of Key Genes in Important Immune Pathways/Cascades in BmβGRP Overexpressed BmN Cells. (**A**–**G**) Relative expression levels of key genes, including BmPPO (**A**), BmPPAE (**B**), BmPelle (**C**), BmMYd88 (**D**), BmCecA (**E**), BmRelish (**F**), and BmCecB (**G**), were assessed by qPCR in BmN cells transfected with pIZT-βGRP or pIZT-Red at 24, 48, and 72 h post-transfection (h.p.t). Among these, BmPPO (**A**) and BmPPAE (**B**) are key genes in the proPO cascade; BmPelle (**C**) and BmMYd88 (**D**) are key genes in the Toll pathway; and BmCecA (**E**), BmRelish (**F**), and BmCecB (**G**) are key genes in the Relish-mediated immune cascade. Data represent mean ± SD from three biologically independent experiments. * *p* < 0.05, ** *p* < 0.01, *** *p* < 0.001, **** *p* < 0.0001.

Previous studies have demonstrated that βGRP-1 can activate downstream immune signaling pathways, including the ProPO cascade and Toll pathway. To investigate whether βGRP-3 possesses similar functions, we overexpressed βGRP-3 and mCherry in BmN cells (BmN-tβGRP and BmN-tmCherry, respectively) and used qPCR to detect the expression of several marker genes associated with immune signaling pathways at 24, 48, and 72 h. These markers included BmPPAE and BmPPO for the ProPO cascade, BmMyD88 and BmPelle for the Toll pathway, BmFADD and BmDreed for the Imd pathway, and BmRelish, BmCecA, and BmCecB for the Relish-mediated immune cascade. The results showed that the BmPPO gene was upregulated at 48 and 72 h in BmN-tβGRP compared to BmN-tmCherry (Figure 5A), while the expression level of BmPPAE did not change significantly (Figure 5B). BmPelle and BmMyD88 were upregulated at 24 and 48 h, respectively, with the overexpression of βGRP (Figure 5C,D). Additionally, BmCecA was upregulated at 24 h in BmN-tβGRP compared to BmN-tmCherry (Figure 5E), while BmRelish and BmCecB were upregulated at 48 h (Figure 5F,G). These results suggest that the ProPO cascade, Toll pathway, and Relish-mediated cascade can be activated by βGRP upregulation, potentially contributing to its antiviral function through the activation of these immune pathways.

## 4. Discussion

In this study, we identified BmβGRP, an important Pattern Recognition Receptor (PRR) of *Bombyx mori*, by its potential antiviral function, as indicated by its significant suppression during BmCPV infection. To elucidate this, we conducted a series of experiments to test the potential antiviral function of BmβGRP. Both transient and stable overexpression of BmβGRP in BmN cells led to a reduction in the expression of the BmCPV VP1 protein, a marker of intracellular viral proliferation, analogous to the VP39 protein in BmNPV [34,35]. Conversely, RNA interference of BmβGRP gene in BmN cells, which stably overexpressed the BmβGRP gene, promoted the intracellular proliferation of BmCPV. These results collectively suggest that BmβGRP functions as an antiviral PRR, aiding *Bombyx mori* in defending against BmCPV infection.

Pattern recognition receptors (PRRs) are specialized receptors that recognize specific pathogen-associated molecular patterns (PAMPs), initiating downstream immune signaling cascades to combat pathogenic microorganisms [36]. Previous studies have established that β-1,3-glucan recognition proteins (βGRPs) specifically bind to β-1,3-glucans via their N-terminal domain. While β-1,3-glucans are predominantly present in the cell walls of bacteria and fungi, their existence in viruses has not been confirmed. Therefore, the N-terminal domain of *BmβGRP-3* is likely a β-1,3-glucan binding domain. However, the functional role of the C-terminal domain in both BmβGRP-1 and *BmβGRP-3* remains unclear. To explore whether the C-terminal domain of *BmβGRP-3* can interact with BmCPV virions, we employed AlphaFold 3 to predict potential interactions between *BmβGRP-3* and various structural proteins of BmCPV, including the capsid shell protein (CSP, VP1), spike protein (VP3), turret protein (TP, VP4), and large protrusion protein (LPP, VP5). The prediction results revealed an interface predicted template modeling (ipTM) score of 0.66 for the interaction between CSP and *BmβGRP-3*, while the ipTM scores for other interactions were below 0.6, indicating a low likelihood of successful prediction. These findings suggest that *BmβGRP-3* may interact with the CSP of BmCPV, potentially triggering downstream antiviral immune signaling cascades.

In our study, we demonstrated that *BmβGRP-3* is capable of activating multiple downstream immune cascades, including the ProPO cascade, the Toll pathway, and the Relish-mediated cascade (Figure 5). These pathways have been well-documented to play critical roles in antiviral defense mechanisms in invertebrates [37,38,39,40,41,42,43]. Consequently, the suppression of pattern recognition receptor (PRR) expression may represent a key viral strategy to circumvent host immune surveillance. Regardless of the specific antigenic determinants that engage PRRs, the activation of these downstream immune cascades can effectively inhibit viral proliferation. Additionally, this mechanism may provide a plausible explanation for the observed phenomena of immune memory in invertebrates [44,45], despite the potential absence of pathogen-specific recognition [46].

Integrating our experimental findings with the above discussion, we can envision an intricate immune battle centered around βGRP between BmCPV and *Bombyx mori*. Upon infection of *Bombyx mori* larvae, BmCPV virions are recognized by βGRPs, which likely interact specifically with the capsid shell protein (CSP) on the viral surface. This recognition initiates the activation of downstream immune cascades, including the ProPO cascade, Toll pathway, and Relish-mediated pathway, collectively mounting a robust antiviral defense in the host. However, to evade this immune response, BmCPV employs a counter-strategy by suppressing the expression of βGRPs, thereby disrupting the activation of these immune pathways and promoting viral proliferation. This proposed mechanism underscores the pivotal role of βGRPs in orchestrating antiviral immunity and highlights the evolutionary arms race between the host and the pathogen, wherein BmCPV has adapted to subvert host defenses by targeting the expression of key pattern recognition receptors (PRRs).

In summary, our study revealed the potential antiviral function of *BmβGRP-3*, which has not been previously reported, and explained why BmCPV decreases the expression level of *BmβGRP-3* during infection. This study provides insights into the host-virus interaction mechanisms evolved over billions of years. Additionally, it offers a new target for developing anti-BmCPV compounds in sericulture and constructing transgenic silkworm strains resistant to BmCPV.

## Figures and Tables

**Table 1:** Primers for qPCR.

Labels of Primer	Primer Pair (5′–3′)	Note
VP1-qPCR-F	ACCATTAACGCTGCTGGTGA	Product’s Length: 116 bp NCBI ID: AY388398.1
VP1-qPCR-R	GCTCCGGCTAGTGGTACATC	
RP49-qPCR-F	CAGGCGGTTCAAGGGTCAATAC	Product’s Length: 213 bp NCBI ID: AY769302.1
RP49-qPCR-R	TGCTGGGCTCTTTCCACGA	
BmRelish-qPCR-F	TTCGGTGGAATGGGTATCAT	Product’s Length: 165 bp NCBI ID: NM_001102465.1
BmRelish-qPCR-R	GCTGAACTTCAAACGCACAA	
BmCecA-qPCR-F	GCCCAGGTGGAAACTCTTCA	Product’s Length: 99 bp NCBI ID: NM_001043997.1
BmCecA-qPCR-R	GCTTGCCCTATGACGGCTAT	
BmCecB-qPCR-F	ATCCTTCGTCTTCGCTCTGG	Product’s Length: 106 bp NCBI ID: D11114.1
BmCecB-qPCR-R	ACGGATGTTCCTGCCCATTT	
BmDredd-qPCR-F	TGTGCGAGGGCACATTTTTG	Product’s Length: 176 bp NCBI ID: AB292816.1
BmDredd-qPCR-R	CCAAGGGCGAGTCAGTTGTT	
BmFADD-qPCR-F	AACAGGTACAAACTGGCGGG	Product’s Length: 142 bp NCBI ID: NM_001202536.1
BmFADD-qPCR-R	TTGTGGATCTGCTCGTTGCT	
BmMyd88-qPCR-F	ACACAGAATTGTTCCCAGGCT	Product’s Length: 128 bp NCBI ID: XM_004921516.5
BmMyd88-qPCR-R	GGCTCTCCGATCTGAACTCG	
BmPPAE-qPCR-F	TGTTTGCTGTCCATGCAACG	Product’s Length: 175 bp NCBI ID: NM_001043367.1
BmPPAE-qPCR-R	TGGCGCAGTAACACAGCATA	
BmPPO-qPCR-F	GCAGCAGCGTAATGGGAATG	Product’s Length: 148 bp NCBI ID: EU569724.1
BmPPO-qPCR-R	CGGATTGTGTTGTTGCCAGG	

**Table 2:** Primers for Construction of Overexpression Plasmids and dsRNA synthesis.

Labels of Primer	Primer Pair (5′–3′)	Note
mCherry-F	GGCGGCCGCTCGAGTCTAGAATGGTGAGCAAGGGCGAGGA	Construct the Overexpression Plasmid pIZT-mCherry-egfp
mCherry-R	TTCGAACCGCGGGCCCTCTAGACGTTACTTGTACAGCTCGTCCATGC	
βGRP-F	AGCGAATTTAAAGCTTGGTACCGAGCTCATGTTCTTCAAAATTATTATAT	Construct the Overexpression Plasmid pIZT-βGRP-egfp
βGRP-R	GGAGAGGGTTAGGGATAGGCTTACCACTAGTAAGTGCATAAACTCTGACG	
dsRNA-Red-F	TAATACGACTCACTATAGGGAAGCTGAAGGTGACCAAGG	Synthesis dsRNA dsRed
dsRNA-Red-R	TAATACGACTCACTATAGGGTGGTGTAGTCCTCGTTGTGG	
dsRNA-βGRP-F	TAATACGACTCACTATAGGGGATAACGGCGAATGGACTGT	Synthesis dsRNA dsβGRP
dsRNA-βGRP-R	TAATACGACTCACTATAGGGGGTCATAACTGGCGGAAGAA	

## Data Availability

The high-throughput sequencing data were deposited in the Bioproject database (https://ngdc.cncb.ac.cn/bioproject/ (accessed on 25 July 2023)) under the Bioproject ID: PRJNA931460.

## Supplementary Materials

The following supporting information can be downloaded at: https://www.mdpi.com/article/10.3390/insects16040431/s1, Figure S1: Transcriptome analysis results in the midgut tissues of silkworms.; Figure S2: Expression patterns of genes involved in the Toll signaling pathway.
