# Supplementary material for: β-1,3-Glucan Recognition Protein Can Inhibit the Proliferation of Bombyx mori Cytoplasmic Polyhedrosis Virus
Source: Insects. 2025 Apr 19;16(4):431. doi: 10.3390/insects16040431 (PMC12028182; doi:10.3390/insects16040431)
Supplement: Supplementary file 1 [file insects-16-00431-s001.zip › insects-3409967-supplementary.pdf]

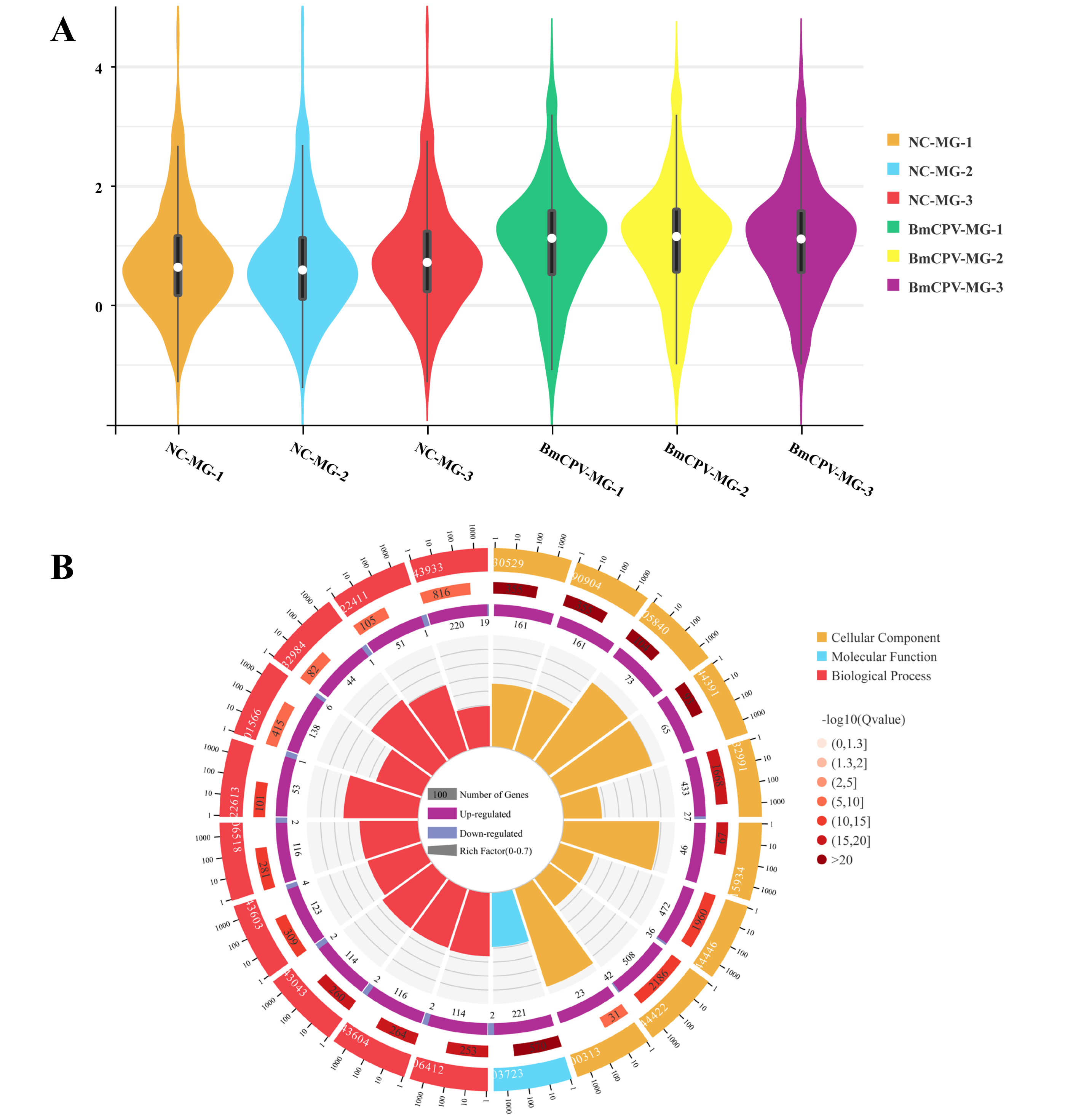

Figure S1. Transcriptome analysis results in the midgut tissues of silkworms. (A) The violin plot illustrates gene expression abundance across the NC-MG and BmCPV-MG groups. (B) Differentially expressed genes are mapped to Gene Ontology (GO) terms under molecular function (MF), cellular component (CC), and biological process (BP) categories. The outermost ring represents the number of differentially expressed genes within each GO term. The first circle displays the GO term IDs and their associated ontologies. The second circle indicates the number of genes enriched within each GO term. The third circle highlights the number of differentially expressed genes, color-coded to indicate up- and down-regulation trends. The innermost circle shows the ratio of differentially expressed genes enriched in each GO term relative to the background genes.

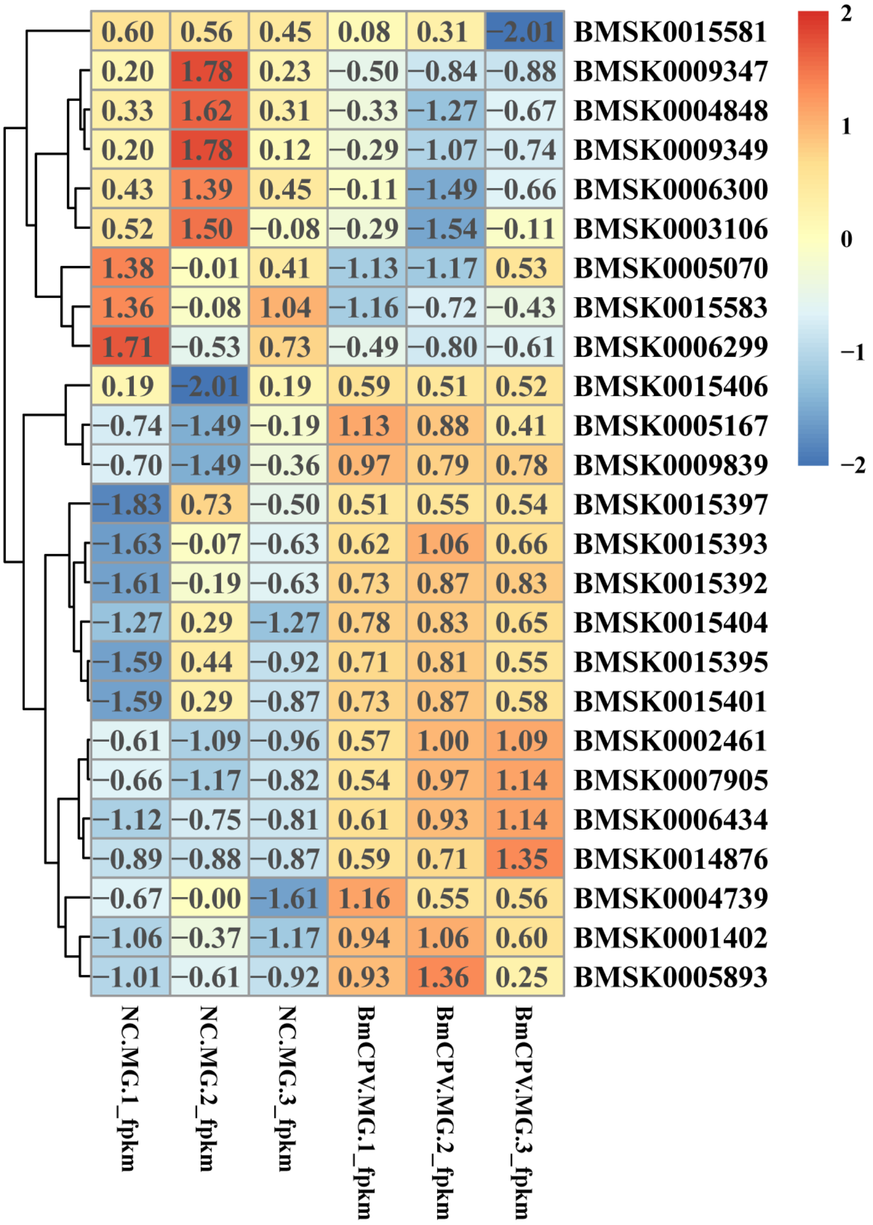

Figure S2. Expression patterns of genes involved in the Toll signaling pathway. A heatmap was generated using R software, with transcriptome data normalized through log2 transformation.
